# Identify the digitalization technology opportunity of low-carbon energy technologies: Using the patent data and collaborative filtering

**DOI:** 10.1371/journal.pone.0309420

**Published:** 2024-09-03

**Authors:** Jie Liu, Wanlin Cai

**Affiliations:** 1 School of Intellectual Property, Nanjing University of Science and Technology, Nanjing, Jiangsu, China; 2 School of Economics, Zhejiang University of Finance and Economics, Hangzhou, Zhejiang, China; Sichuan Agricultural University, CHINA

## Abstract

The digitalization of low-carbon energy technologies (LCET) provides important technical support for the transition to a greener energy system. Digitalization addresses the phenomenon of the growing application of information and communications technologies (ICT) across the economy, which is regarded as the technology convergence between ICT and other technologies. Scholars have revealed the signs that LCET and ICT are becoming increasingly interlinked, which raises the challenges for predicting and identifying the technology opportunities for innovations in the converged technology area. To address the challenges, this paper proposes a collaborative filtering approach to identify the digitalization technology opportunity of low-carbon energy technologies using patent classification and patent citation information. We applied the proposed collaborative filtering approach using a large LCET patent dataset derived from the United States Patent and Trademark Office (USPTO). The results indicate that the proposed method can effectively identify digitalization technology opportunities of LCET, and the current LCET digitalization technology opportunities identified based on this approach are mainly concentrated in the Energy storage field. The advantages of the proposed approach are that its underlying data are more readily available and its technical complexity is relatively lower, and thus, more replicable for other technology fields.

## Introduction

The pervasiveness and integration of digital technologies into the economy and society have profoundly impacted social life and emerged as a crucial driver for high-quality economic development. Digitalization addresses the phenomenon of the growing application of information and communications technologies (ICT) across various sectors of the economy [1]. In the energy system, rapid digitalization, such as the development of smart grids and the Energy Internet, provides critical technical support for the transition to a greener economy [2, 3]. As the time window for limiting global warming to a manageable level is closing [4], and considering the need for cross-domain integration, energy digitalization–the technology convergence between energy technologies, especially Low-Carbon Energy Technologies (LCET), and ICT–has attracted broad attention [1, 3, 5]. Technology convergence refers to the phenomenon where technology domains overlap, and it has been viewed as a significant driver of technology change along with the development of increasingly complex products [6, 7].

Following and identifying the research and development (R&D) directions for LCET digitalization is of great strategic importance for both firms and policy-makers. For firms in LCET and ICT industries, accurately capturing the technology convergence trajectory is crucial for adapting to the changing competitive landscape [7]. For policy-makers, it is essential to strategically plan innovation policy instruments to accelerate technology convergence, which can impact a country’s competitiveness in the technology markets [5], as well as accelerate the decarbonization of the energy system [1]. Although the convergence of ICT with different sectors, such as broadcasting, entertainment, and biotechnology, has been the subject of numerous studies using patent data [8, 9], the study of ICT convergence with LCETs using patent data has received little attention, with few exceptions, which show the signs of technology convergence between LCET, such as solar PV, wind, and energy storage technologies and ICT [3, 5]. Given that LCET and ICT are becoming increasingly interlinked, previous studies fail to provide specific and practical technology opportunities for LCET digitalization. It is still difficult to make decisions on the R&D direction of digitalization, and thus, it raises challenges for identifying digitalization technology opportunities in the converged technology fields [5].

To address the challenges, this paper presents an innovative approach to identifying digitalization technology opportunities in LCET by utilizing an adapted collaborative filtering method incorporating patent classification and patent citation data, from the perspective of technology convergence. The contribution of this paper is twofold: First, given the challenges of identifying and capturing the opportunity window of technology change derived from the digitalization transformation in LCET, this paper serves as an important supplement to existing research. Second, at the methodology level, the adapted collaborative filtering method proposed in this paper has advantages such as low technical complexity and novel recommendations. Compared to text-mining-based methods that may rely on researchers’ subjective judgment, this method has a stronger repeatability. Besides, while collaborative filtering has been applied in firm-level technology opportunity identification, this paper expands its application to the industry level, and thus broadens the application scope of this method.

Specifically, we initially empirically validate the effectiveness of the proposed collaborative filtering approach in identifying historical digitalization technology opportunities based on the LCET patents applied in the period 2011–2015. Subsequently, leveraging the LCET patents applied in the period 2016–2020, we dive deeper into current LCET digitization technology opportunities. Our findings reveal that the LCET digitalization technology opportunities identified by our method are predominantly concentrated in the field of Energy storage, accounting for over 50% of the identified LCET CPC codes. Policy implications could be derived from the results.

The rest of the paper is organized as follows: The “Literature Review” section shows the literature review; the “Methodology” section provides the details of the proposed method. The identification of digitalization technology opportunities in LCET domains is provided in the section “Empirical analysis: the LCET case”, the “Discussion” section shows the discussion, and the “Conclusions” section provides the conclusions.

## Literature review

### The digitalization of LCET

The challenges to mitigate the influence of human-induced climate change have led to significantly increasing efforts to stimulate eco-innovations, i.e., innovations that contribute to reducing environmental burdens [10, 11]. Along with the development and pervasiveness of digital technologies, many scholars have reached a wide consensus that eco-innovations have been linked to the technical change in the ICT domain [11, 12]. Digitalization describes the growing application of ICT across the economy [1]. As a notable example, digitalization in the energy system is having profound impacts on both energy demand and supply, which could improve energy efficiency in the whole energy sector [1]. In this paper, we focus on the digitalization of one kind of specific eco-innovative technological solutions that has been regarded as the key to the transition to a sustainable economy [10, 13, 14], namely low-carbon energy technologies (LCET) that refers to technologies aimed at reducing greenhouse gas emissions, energy consumption, environmental impacts, as well as contribute to redesigning the global energy system [15, 16].

The rapid digitalization in the energy sector, particularly the LCET domains, such as renewable energy production and energy storage domains, provides a promising pathway toward a sustainable energy system–one characterized by higher resilience and flexibility [5, 17, 18]. Along with this, the significance of emerging digital technologies, such as blockchain [19], energy big data, and cloud computing [20] has been recognized. Meanwhile, scholars have provided empirical evidence that justifies energy digitalization for environmental sustainability. For example, Shi et al. [21] find that energy digitalization exhibits a statistically significant ability to enhance regional carbon productivity in China.

However, the digitalization processes of LCET are not always linear. For example, Kangas et al. [5] proposed that the immature nature of solar PV technology shadowed its digitalization development. This shadowed digitalization trend is thought to be continued since there is considerable improvement potential in energy conversion efficiency and cost efficiency in basic material technologies. Meanwhile, the depth of digitalization may be not equal across different parts of a field [5]. In this regard, to foster comparative advantages in the information era, LCET firms need to follow and predict ICT developments and identify opportunities for digitalization development, for which the underlying theory is built on the more general technology convergence literature [5].

### Technology convergence and monitoring LCET digitalization using patent data

Technology convergence has long been recognized as an important driver of technology change [6, 7], which denotes the overlap between hitherto separate technology domains [22]. The concept of technology convergence naturally matches the digitalization dynamics well. According to the definition of digitalization, i.e., the growing application of ICT across the economy, the LCET digitalization processes could be regarded as the convergence between ICT and LCET technologies [3, 5].

Following the main strand of convergence studies, this study monitors the LCET digitalization dynamics using patent data [22]. Patent data, which is regarded as the important carrier of technology innovation outputs, has been employed in technology evolution and convergence analysis in several previous studies [7, 10, 23–26]. Patent co-classification analysis is the most common patent-based technology convergence measurement method [5]. Patent co-classification refers to different patent classification codes being assigned to a single patent document, which denotes that the invention holds the technical features of different technology fields indicated by different patent classification codes. The increasing co-classifications of previously separated patent classification codes imply technology convergence [5]. Similarly, technology convergence can also be identified by the rise of patent citations between different technology domains [7, 9, 27, 28].

Note that compared to the co-classification, patent citation measurement is thought to be more appropriate to describe the stretching process between different domains, rather than to the actual technology convergence event that signifies the creation of hybrid new technology [7]. Stimulated by this argument, in this paper, we identify the digitalization process, which is represented as the ICT convergence, based on patent citation data to capture the boundary-blurring process between technology domains. Besides, Caviggioli [7] proposed that the cross-citations can work as a predictive factor of the co-classification event. We posit that identifying digitalization technology opportunities based on patent citation information will be of higher farsightedness.

In terms of the application of patent-based technology convergence analysis in the theme of "digitalization", although the research on ICT convergence based on patent data has received attention for a long time, few studies focus on the convergence between ICT and LCET. To our knowledge, only a few exceptions have analyzed the ICT convergence trend of solar PV, wind, and energy storage fields using patent co-classification data [3, 5]. Given the rapid development in basic technologies, as well as the shadowed digitalization processes, these available patent-based LCET digitalization studies suggest the importance of identifying LCET digitalization opportunities.

### Collaborative filtering and its application in technology opportunity identification

Collaborative filtering is one of the most widely used recommendation methods, which aims to recommend items that are suitable for a target user based on the information of the user’s preference and the historical purchasing data [29]. The first automotive collaborative filtering system, known as GroupLens [30], aims to recommend news articles to target users. Its logic is rooted in the assumption that if a particular group of users has had consistent preferences for news in the past, their preferences will remain consistent in the future. GroupLens collects user preferences through rating, i.e., users rating the articles they have read (ratings range from integers 1 to 5, with higher scores indicating greater user preference for the article). The system then calculates the similarity of preferences among users, and selects a group of users with high similarity to the target user to predict the target user’s preference for new articles.

Compared to other recommendation systems, collaborative filtering has several advantages. First, collaborative filtering does not require understanding the item itself, as it does not depend on the item information. Second, the collaborative filtering technique can recommend unexpected items because this technique is based on other users’ historical data [29, 31, 32]. Collaborative filtering is known for its simplicity and effectiveness [29], and has been applied in many studies, such as facilitating knowledge collaboration between developers [33] and identifying new R&D ideas [34].

Technology opportunities are a set of opportunities with the possibility of technological progress [35]. Identifying technology opportunities has a profound impact on industries’ and firms’ innovation [36, 37]. Technology opportunity discovery (TOD) refers to discovering and selecting the best opportunities for the industry or firm from a large amount of data [29, 36]. It can supplement the subjective ideas of traditional researchers and engineers, ultimately enhancing innovation efficiency [36]. Collaborative filtering recommendations have been utilized to identify technology opportunities. Park et al. [29] developed a firm-level technology opportunity identification method based on patent classification and collaborative filtering, the effectiveness of which has been verified in empirical analysis. In this paper, based on the method of Park et al. [29], we construct an adapted collaborative filtering method for identifying industry-level digitalization technology opportunities.

## Methodology

**Fig 1:**
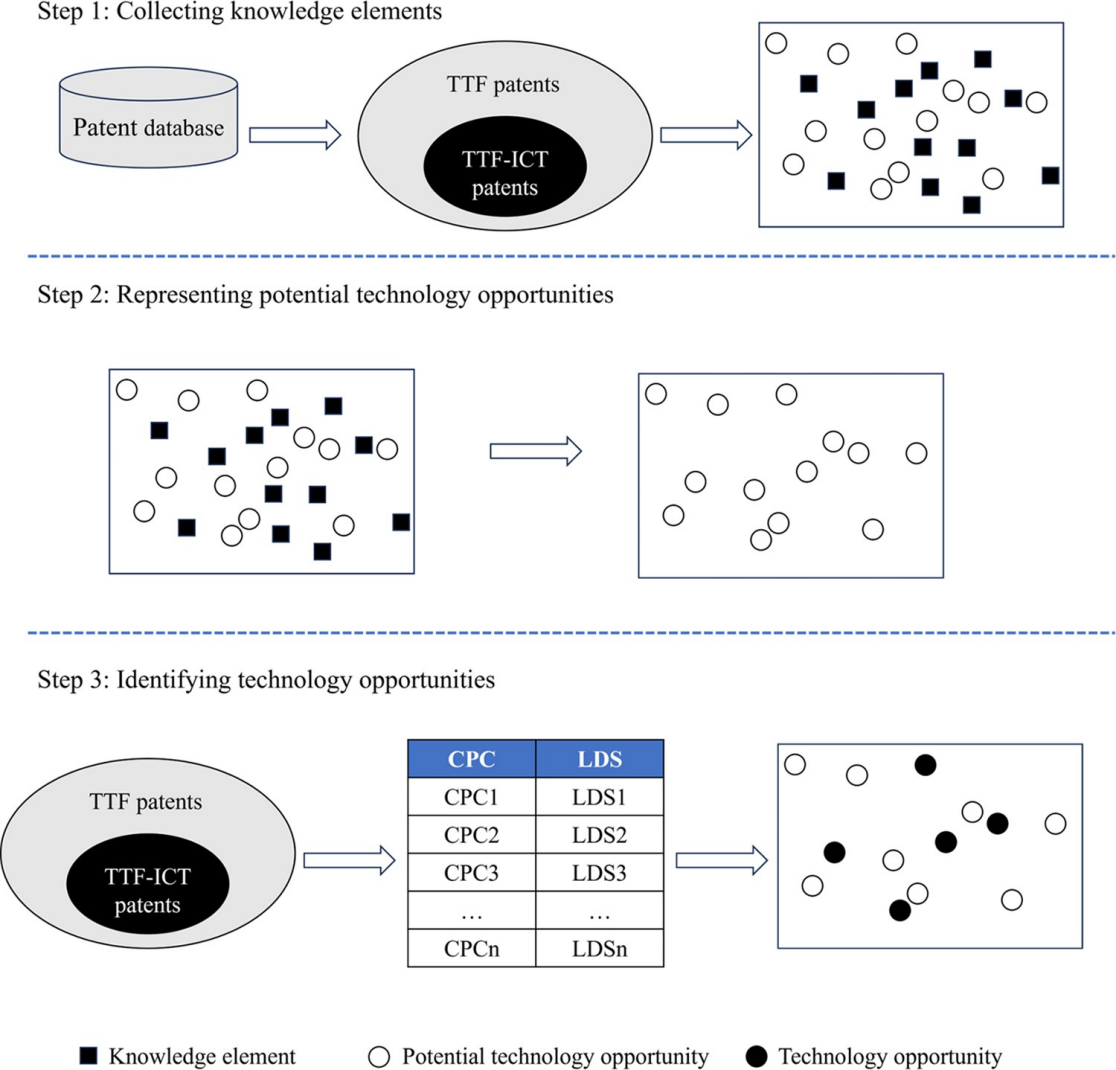
The framework for identifying digitalization technology opportunities based on collaborative filtering.

In this paper, a methodology for identifying the technology opportunities that have a high potential for integrating digital technology solutions is suggested, based on the industry’s current technological knowledge base. Following the prior work of Park et al. [29], this paper utilizes a set of patent classification codes to represent the technological knowledge base of the focal domain. In specific, the Cooperative Patent Classification (CPC) codes are employed to denote knowledge elements. It then recommends the potential classification codes using a collaborative filtering technique. The methodology, with its simple and automatic implementation process, is highly replicable in other technology domains. The methodology proposed in this paper consists of three major steps: (1) Collecting knowledge elements, (2) Representing potential technology opportunities, and (3) Identifying technology opportunities. The proposed implementation process is illustrated in Fig 1.

### Collecting knowledge elements

A knowledge element refers to a self-standing embodiment of a core concept in a distinct scientific or engineering principle within a certain technology field [36, 38]. The CPC codes of patents in the target technology field (TTF) are used to denote the knowledge elements in that field and are referred to as CPC_TTF_ in the remainder of this paper. Based on the logic of collaborative filtering, then, it is necessary to calculate the similarity between knowledge elements. In this paper, since the convergence process is measured using patent citation information, we propose the citation-based measurement of similarity between CPC_TTF_ to capture the logical consistency.

Specifically, consider *N*_*1*_ as the number of TTF patents and *L* as the number of unique CPC_TTF_. Then, the binary *N*_*1*_*⊆L* matrix *A* is defined as *A*_*il*_ = 1 if TTF patent *i* contains CPC_TTF_ *l*. Similarly, consider *K* unique CPCs which are assigned to *N*_*2*_ patents cited by TTF patents, i.e., k unique CPC_REF_, the binary *N*_*2*_*⊆K* patent-CPC matrix *B* can be defined as *B*_*jk*_ = 1 if the cited patent *j* contains CPC_REF_ *k*. The matrices *A* and *B* are coupled via citation relationships, which is represented as a binary *N1 × N2* citation matrix *M*.

The *l*^*th*^ row of the matrix *A*^*T*^*M* shows the number of citations from CPC_TTF_ *l* to any cited patent *j*. In the same way, the *l*^*th*^ row of the matrix O = *A*^*T*^*MB* gives the number of citations from CPC_TTF_ *l* in TTF patents to any CPC_REF_ *k* in the cited patents.

Then, to avoid the over-distribution of citations which can disturb the effectiveness of collaborative filtering, following Park et al. [29], we use fuzzy logic to transform the above matrix values into five-point scale values, which is given as:

$${FO}_{l,k}=\left\{ \begin{array}{l} 5\times {\left(1+{O_{l,k}}^{-a}\right)}^{-b},\;O_{l,k}>0\\ \;0\;,\;O_{l,k}=0 \end{array}\right.$$ (1)

where *O*_*l*,*k*_ is the number of citations from CPC_TTF_ *l* to CPC_REF_ *k*, and *FO*_*l*,*k*_ is the fuzzy logic transformed citation numbers. In this way, the cited technology portfolio (CTP) corresponding to any CPC_TTF_ *l* is given as:

$${CTP}_{l}=[{FO}_{l,1},{FO}_{l,2},\ldots ,{FO}_{l,k}]$$ (2)

Finally, the cosine similarity coefficient is used to calculate the similarity (CSTTF) between CPC_TTF_. The formula is as follows:

$$CSTTF({CPC}_{TTF,i},{CPC}_{TTF,j})=\frac{\sum_{n=1}^{K}{FO}_{i,n}\times {FO}_{j,n}}{\left|{CTP}_{i}|\times |{CTP}_{j}\right|}$$ (3)

### Representing potential technology opportunities

In this paper, CPC codes that have not been integrated with ICT solutions are represented as potential technology opportunities. To identify these potential technology opportunities, TTF patents that cite ICT patent(s) or could be identified as ICT patents, referred to as TTF-ICT patents, are considered as instances of digitalization. We posit that the TTF patents citing ICT patent(s) imply the stretching process to the ICT domain, while if a TTF patent is also an ICT patent, it could be regarded as a complete digitalization event. Consider a binary vector D of length *N*_*1*_, where D_*i*_ is 1 if patent *i* is TTF-ICT patent. The *l*^*th*^ value of DF = A^T^D represents the number of TTF-ICT patents in CPC_TTF_ *l*. Then, using the fuzzy logic mentioned above, we transform it to obtain the digitalization score (DS) of each CPC_TTF_:

$$DS=[{FDF}_{1},{FDF}_{2},\ldots ,{FDF}_{l}]$$ (4)

where *FDF*_*l*_ is the transformed *l*^*th*^ DF value. In this paper, we define CPC_TTF_ with the DS of 0, i.e., patents in these CPC_TTF_ are not TTF-ICT patents, as potential technology opportunities. This setting implicitly assumes that all the knowledge elements represented by CPC_TTF_ would eventually be integrated with digital technology solutions for new inventions.

### Identifying technology opportunities

Based on CSTTF and DS, we can calculate the latent digitalization score (LDS) of each potential technology opportunity, which is given as follows:

$$LDS({CPC}_{TTF,i})=\sum_{j=1}^{L}CSTTF\prime ({CPC}_{TTF,i},{CPC}_{TTF,j})\times {DS(FDF}_{j})$$ (5)

where CSTTF’ denotes the modified CSTTF, in which the similarity values below a threshold are set to 0. According to the logic of collaborative filtering, the higher the LDS (CPC_TTF,i_), the greater the likelihood CPC_TTF,i_ is integrated with digital solutions in subsequent stages.

## Empirical analysis: the LCET case

### Data source

The patent dataset used in this analysis is derived from the PatentsView platform (https://patentsview.org/download/data-download-tables) in June 2024, which contains the granted patents in the United States Patent and Trademark Office (USPTO) since 1976. The application year is used as the indicator of time for each invention. The reason for this setting is that the application date is closer to the inventions’ actual creation time, which facilitates reflecting the temporal technology dynamics more accurately [39]. Besides, to focus the analysis on high-quality technology activities, only the utility patents are considered in this paper (for a similar setting, see [40]).

In this paper, the CPC codes are employed to identify LCET patents. Following Park et al. [29], the CPC main groups are used to denote the knowledge elements. The CPC system is divided into nine sections, A-H and Y, which are further subdivided into classes, subclasses, main groups, and subgroups [41, 42]. Table 1 shows an example of the CPC structure. The CPC system was developed by the European Patent Office (EPO) and USPTO to harmonize patent classifications and to replace the former European Classification System (ECLA) and U.S. Patent Classification (USPC) system. The CPC system is similar to the International Patent Classification (IPC) but is more detailed and comprehensive [43]. A significant difference between CPC and IPC is that CPC contains the “Y” Section. The CPC codes in the “Y” section do not indicate separate technological classes but are additional tags attached to patents by examiners to tag some special technical subjects. The "tags" corresponding to the LCET are in CPC subclass "Y02E". Note that CPC in the “Y” section are not treated as knowledge elements in this paper, and are only used to identify LCET patents.

**Table 1:** Example of the CPC system structure.

Hierarchy	Codes	Description
Section	H	Electricity
Class	H01	Electric elements
Subclass	H01C	Resistors
Main group	H01C 10	Adjustable resistors
Subgroup	H01C 10/02	Liquid resistors

To identify the ICT patents, the IPC code list of ICT patents employed by Kangas et al. [5] and Zhang et al. [3] is used (Table 2 provides the IPC code list of ICT patents). We then use the CPC to IPC concordance table (https://www.cooperativepatentclassification.org/cpcConcordances, accessed in June 2024) to identify the corresponding CPC codes for ICT patents. Patents that are assigned with those CPC codes are identified as ICT patents. The LCET-ICT patents are defined as LCET patents that cite ICT patents or that can be identified as ICT patents. An LCET-ICT patent is regarded as an instance of LCET digitalization.

**Table 2:** The IPC code list of ICT patent.

ICT Technology	IPC
Telecommunications	G01S, G08C, G09C, H01P, H01Q, H01S 3/ (025,043,063,067,085,0933,0941,103,133,18,19,25), H01S 5, H03B, H03C, H03D, H03H, H03M, H04B, H04J, H04K, H04L, H04M, H04Q
Consumer electronics	G11B, H03F, H03G, H03J, H04H, H04N, H04R, H04S
Computer, office machinery	B07C, B41J, B41K, G02F, G03G, G05F, G06, G07, G09G, G10L, G11C, H03K, H03L
Other ICT	G01B, G01C, G01D, G01F, G01G, G01H, G01J, G01K, G01L, G01M, G01N, G01P, G01R, G01V, G01W, G02B6, G05B, G08G, G09B, H01B11, H01J (11/,13/,15/,17/,19/,21/,23/,25/,27/,29/,31/,33/,40/,41/,43/,45/)

*Source*: Kangas et al. [5]

### The overall analysis of LCET digitalization

**Fig 2:**
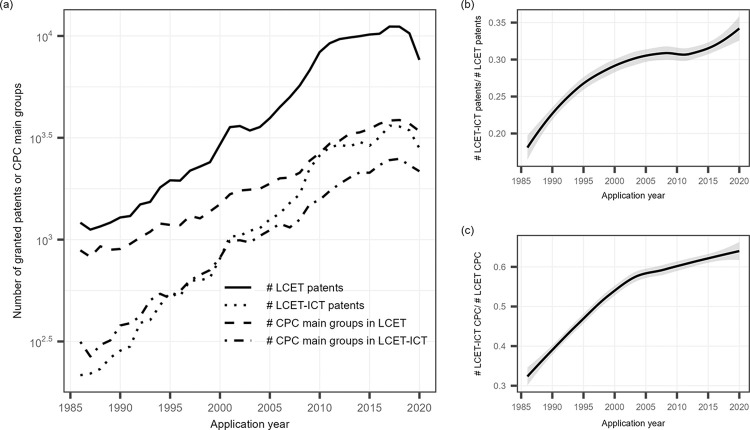
Overall digitalization trend of LCET during 1986–2020. (**a)** presents the annual application number of granted LCET and LCET-ICT patents, as well as the number of annual unique CPC main groups in corresponding LCET and LCET-ICT patents during 1986–2020 **(b)** presents the share of granted LCET-ICT patents in the LCET patents applied during 1986–2020 **(c)** presents the share of unique CPC main groups of granted LCET-ICT patents in the unique CPC main groups of granted LCET patents applied during 1986–2020.

This section provides a description of the LCET innovation and digitalization dynamics over the period 1986–2020. In the period 1986–2020, there were 173,486 granted LCET patents that were identified through the “Y02E” CPC tags, of which 52,709 were LCET-ICT patents. Fig 2(A) shows the evolution of the annual application of granted LCET and LCET-ICT patents, on a semi-log axis, indicating the exponential growth in the invention of LCET and LCET-ICT. However, compared with the growth rate of LCET inventions, the growth rate of LCET-ICT inventions is relatively slow. Thus, in Fig 2(B), one can find a declining trend in the growth rate of the proportion of LCET-ICT patents during 1986–2010. The growth rate of the proportion of LCET-ICT patents increased after 2010, which could partly be explained by the decreased growth rate of LCET patent applications.

Similarly, the number of unique CPC codes in LCET and LCET-ICT patents show an exponential growth trend, while the growth rate of the number of CPC codes in LCET-ICT patents is not as fast as that in LCET patents after 2004, which also leads to a decline in the growth rate of the proportion of CPC codes in LCET-ICT patents after 2004 (see Fig 2(C)). In other words, the overall digitalization degree of LCET has not linearly improved alongside the development of LCET technology. It is necessary to further explore the opportunities and expand the scope of technology convergence between LCET and ICT domains.

### Constructing CPC citation similarity matrix

The collaborative filtering proposed in this paper involves several parameters, including the threshold of the similarity to determine the neighbors of potential technology opportunities and the number of selected technology opportunities based on the LDS ranking. To determine the parameters, the dataset of LCET patents applied in the period 2011–2015 is employed to identify historical digitalization technology opportunities. The parameters are calibrated based on the accuracy of the technology opportunity identification, which is calculated by involving the LCET patents applied in the period 2016–2020. The calibrated parameters are then employed to identify the current digitalization technology opportunities, based on the dataset of LCET patents applied in the period 2016–2020.

To identify the historical digitalization technology opportunities, first, a total of 48,821 granted LCET utility patents applied in the period 2011–2015 are identified based on “Y02E” CPC tags. Then, the granted patents cited by those LCET patents are collected.

The CPC main groups are employed to represent the knowledge elements. The CPC main groups in LCET patents (CPC_LCET_) and cited patents (CPC_REF_) are used to construct the matrix O mentioned in the Methodology section. LCET patents that do not cite other granted utility patents and patents having no CPC information are excluded when constructing matrix O. Based on the 2011–2015 patent data, we construct the matrix O containing 5,011 rows (denoting CPC_LCET_) and 7,701 columns (denoting CPC_REF_). To rescale matrix O into five-point-scale values, following Park et al. [29], the parameters in the fuzzy logic algorithm are set as a = 2 and b = 1. The rescaled matrix O, then, is used to measure the similarity between CPC_LCET_, i.e., CSLCET.

### Measuring the latent digitalization score

The digitalization score (DS) of each CPC_LCET_ is defined as the fuzzy logic rescaled LCET-ICT patent number for each CPC_LCET_, and we define CPC_LCET_ that did not appear in LCET-ICT patents in the analyzed time frame as potential digitalization technology opportunities. Following the principle of collaborative filtering, the next step is to measure the LDS, which is achieved by considering the DS of the neighbors of each potential digitalization technology opportunity and is given as:

$$LDS({CPC}_{LCET,i})=\sum_{j=1}CSLCET\prime ({CPC}_{LCET,i},{CPC}_{LCET,j})\times {DS(FDF}_{j})$$ (6)

The higher the LDS, the more likely it is that CPC_LCET,i_ will be used in LCET-ICT patents in subsequent inventions. Note that as long as the vectors corresponding to the two CPC_LCET_ overlap, the similarity between the two CPC_LCET_ is not 0. To identify neighbors with higher similarity to the focal CPC_LCET_, we set a threshold of similarity, and the similarity values less than the threshold in the CSLCET are set as 0. In this way, the LDS will be calculated based on the DS of neighbors with high similarity to the focal CPC_LCET_.

### Accuracy of historical opportunity identification

The CSLCET constructed based on LCET patents with application years from 2011 to 2015 contains a total of 5,011 CPC main groups, of which 1,430 did not appear in LCET-ICT patents. That is, historical potential technology opportunities encompass 1,430 CPC main groups. To calculate the LDS of these 1,430 CPC main groups, we set the value of the similarity threshold *s* ranging from 0 to 1 with the interval of 0.02, and then the value of the selected technology opportunities number (parameter *n*) ranging from 20 to 200 with the interval of 5. LCET patents applied in the period 2016–2020 are collected to identify whether patents in those CPC are used in LCET-ICT patents in the subsequent inventions. The proportion of the top *n* CPC main groups with the highest LDS scores that are involved in LCET-ICT inventions in 2016–2020 is regarded as the accuracy of the digitalization technology opportunity identification.

**Fig 3:**
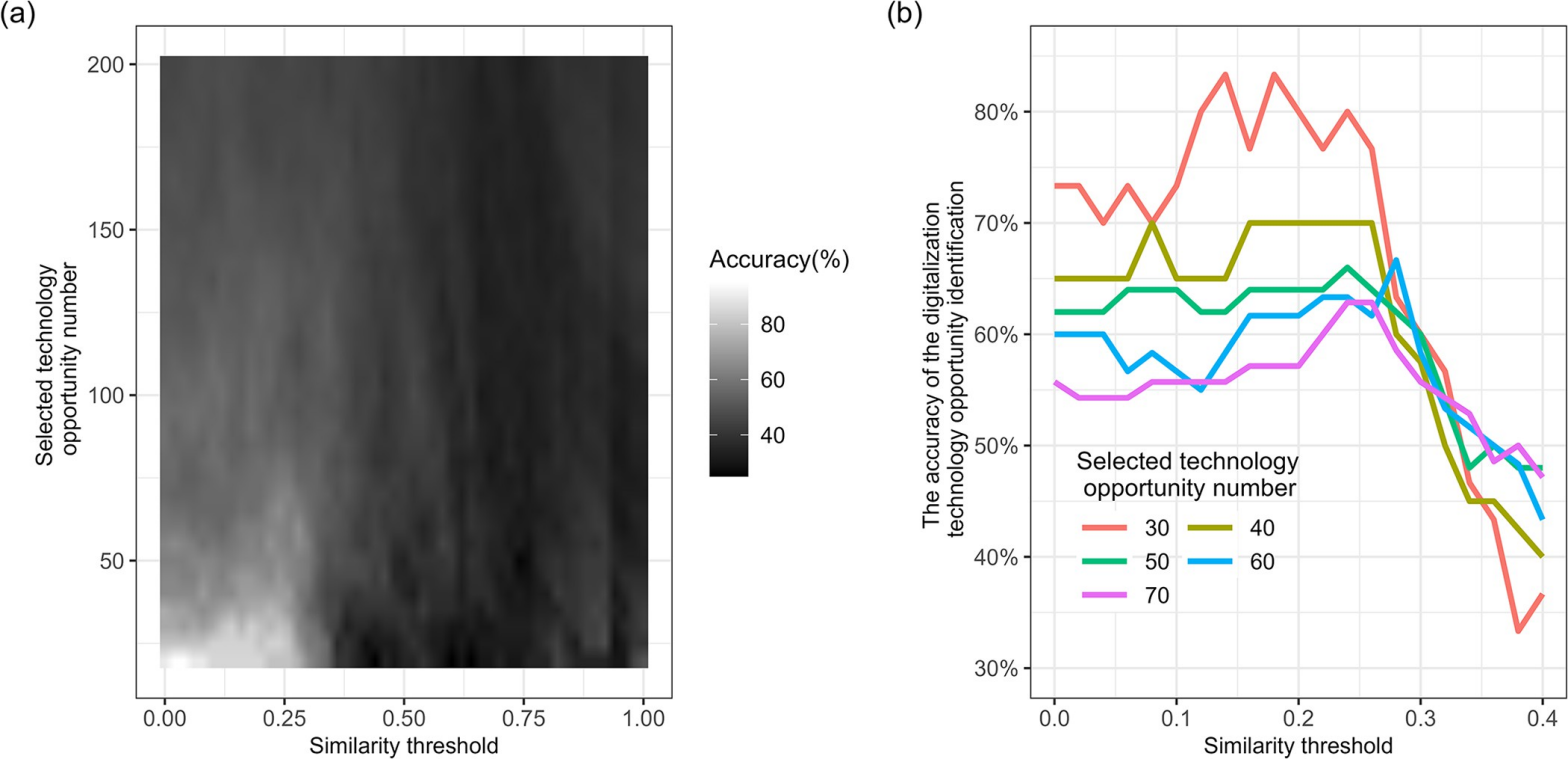
The accuracy of the digitalization technology opportunity identification based on granted LCET patents applied in the period 2011–2015. **(a)** presents the accuracy under different parameter combinations, in which the similarity threshold ranges from 0 to 1, and the selected technology opportunity number ranges from 20 to 200 **(b)** presents the accuracy under different parameter combinations, in which the similarity threshold ranges from 0 to 0.4, and the selected technology opportunity number is set as 30, 40, 50, 60, and 70.

Fig 3 shows the accuracy of technology opportunity identification under different parameter combinations, from which we can see that the accuracy decreases as the two parameters increase. Considering the accuracy and the number of identified digitalization technology opportunities, we set 0.18 and 30 as the values of parameters *s* and *n* respectively. The accuracy in this setting is about 83.3%, which is significantly higher than the digitalization share (around 34.5%) of the total 1,430 CPC main groups in 2016–2020 (the Two Proportions Z-test p-value is less than 0.001).

### Current digitalization technology opportunity identification

After determining the values of s and n, we once again identify the current digitalization technology opportunity of LCET based on the patent data applied in the period 2016–2020. When limiting the patent application years to 2016–2020, a total of 50,403 granted LCET patents are identified with the CPC "Y02E" tags. The CSLCET constructed based on the aforementioned LCET patents contains a total of 5,421 CPC_LCET_, of which 1,351 did not appear in the LCET-ICT patents. We then set CSLCET values less than 0.18 as 0, and calculate the LDS values for the 1,351 CPC main groups.

We also assign the technology opportunities to the specific LCET fields based on the LCET patent data applied during 2016–2020. This assignment focuses on a set of 11 distinct LCET fields identified by “Y02E” CPC tags: Geothermal (Y02E10/1), Hydro (Y02E10/2), Ocean (Y02E10/3), Solar thermal (Y02E10/4), Solar PV (Y02E10/5), Wind (Y02E10/7), Energy storage (Y02E60/1), Hydrogen (Y02E60/3), Fuel cells (Y02E60/5), Clean combustion (Y02E20), and Non-fossil fuel (Y02E50). Patents containing multiple Y02E tags that indicate different focal LCET fields are counted repeatedly for each field. For example, if one patent has two Y02E tags, Y02E10/5 and Y02E10/7, this patent would be recorded as one solar PV patent and one Wind patent. However, if one patent has two Y02E tags for one focal field, such as Y02E10/541 and Y02E10/542, this patent would only be recorded as one Solar PV patent. One issue that arose during the above data processing is that some patents may be classified in coarse CPC codes, indicating that these patents are multipurpose. In this paper, the coarse CPC codes include Y02E10/00 (Renewables excluding Non-fossil fuel), Y02E10/60 (Solar thermal and PV), and Y02E60/00 (Enabling technologies). Patents classified under coarse CPC codes are split, once into each related focal LCET field. The field of a certain technology opportunity is defined as the field with the highest proportion of patents.

The CPC main groups with the top 30 LDS are shown in Table 3. According to the distribution of technology opportunities, the Energy storage field holds the largest part of digitalization technology opportunities, i.e., 16 of the 30 identified CPC main groups. Additionally, the LCET digitalization technology opportunities are mainly concentrated in the following CPC sections: "B. Performing operations; transporting", "C. Chemistry; metallurgy", and "F. Mechanical engineering; lighting; heating; weapons; blasting".

**Table 3:** CPC main groups with top 30 latent digitalization score.

LCETs	Digitalization technology opportunities
Clean combustion	F01N2570, F02G2254, F02P13, F23D2203, F23D17
Energy storage	C08J2205, B01D9, D04H1, C03B19, C08J2427, C22C2026, C03B2201, A62C99, D01F2, C08B17, B02C17, F01P2060, C08L51, B29K2081, D10B2201, B82Y35
Fuel cells	B60K8, C08J2451, A62C99, B29K2081
Hydrogen	B29K2077, C01B2210, A62C99
Non-fossil fuel	C10B31, C08B16, D01F13, C10G21, F02P13, C08B17, D10B2201
Solar PV	C07F3

**Fig 4:**
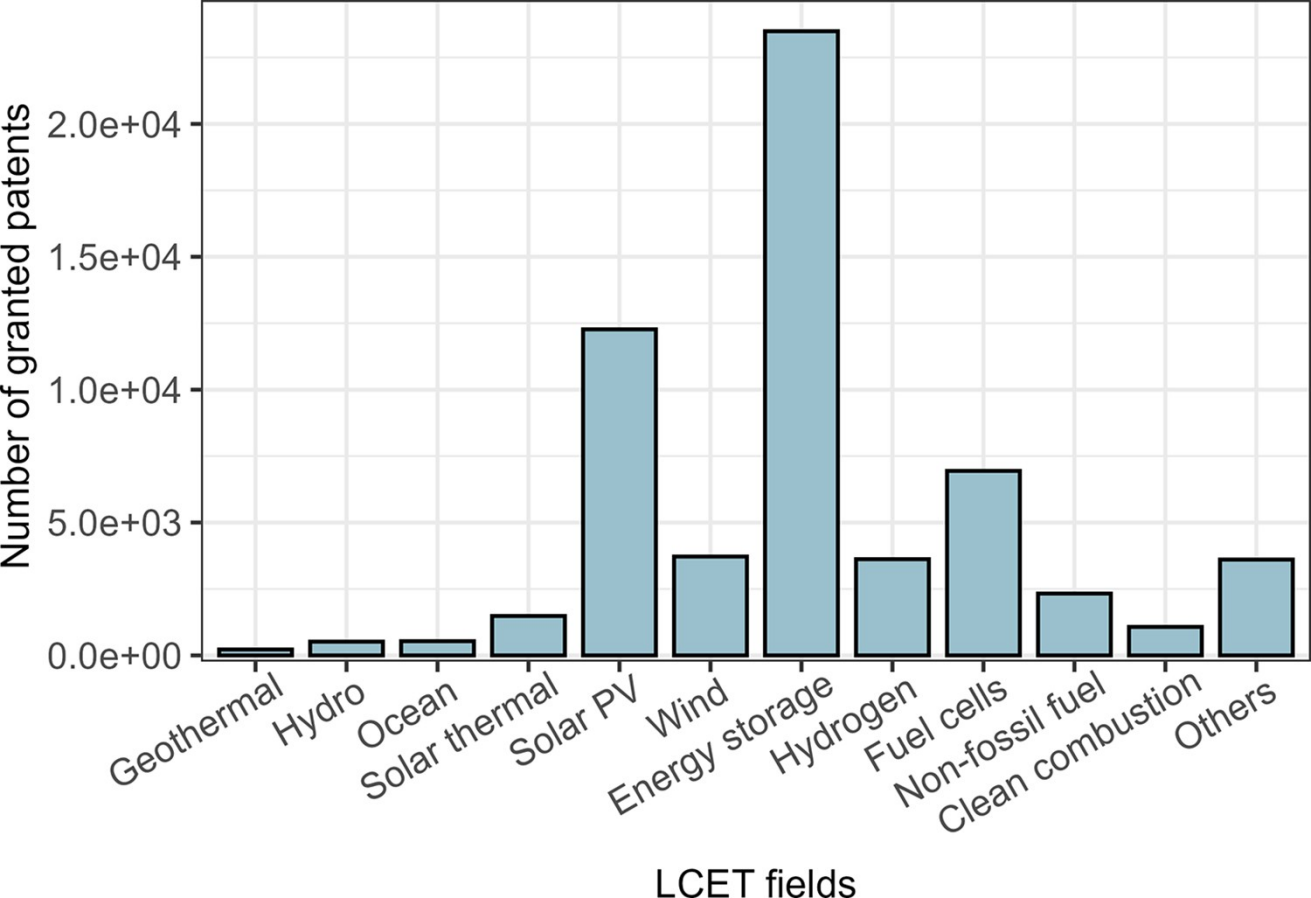
Distribution of granted LCET patents applied in the period 2016–2020 among different LCET fields.

Fig 4 presents the number of granted patents applied in the period 2016–2020 among different LCET fields. Fig 4 shows that more than 20,000 granted LCET patents filed during the focal time window are in the Energy storage field, which is significantly higher than other LCET fields. Given the large number of inventions, it is reasonable to expect considerable digitalization technology opportunities in the Energy storage field. However, the invention volume could only partly explain the distribution of digitalization technology opportunities. Comparing Fig 4 and Table 3, it can be observed that although the number of granted Solar PV patents is the second highest among all LCET fields, there is only one opportunity in the Solar PV field in Table 3. We posit that the nature of technological inventions in the Solar PV field, specifically, that technological improvements might mainly rely on the development of material science, could explain this result.

Table 4 presents the LDS value for each technology opportunity, along with the typical CPC code of LCET-ICT patents (CPC_LCET-ICT_) that exhibits a high degree of LDS contribution to each technology opportunity. Additionally, it lists the typical ICT CPC codes referenced by LCET patents in these CPC_LCET-ICT_, indicating potential digital technology solutions for inventions within each technology opportunity. For example, the technology opportunity "F23D2203” that indicates “Gaseous fuel burners” closely resembles "F23D14” (Burners for combustion of a gas). The ICT patents cited by LCET patents in "F23D14” are mainly in the fields like “G01F23” (Indicating or measuring liquid level or level of fluent solid material). One exemplary patent in " F23D2203” (primarily in the clean combustion field) is titled “Fuel combustion system with a perforated reaction holder”, which provides a solution for holding a combustion reaction that produces very low oxides of nitrogen. The invention involves a fuel and oxidant source to output and mix them into a combustion volume, and a perforated reaction holder with aligned perforations to hold the combustion reaction. The application of digital technologies, such as measuring the level of material, could potentially further enhance the combustion process.

**Table 4:** Information about the CPC main groups with the top 30 LDS value.

CPC main group	LDS	Typical CPC_LCET-ICT_	Typical ICT CPC cited by CPC_LCET-ICT_
F01N2570	867.4774	F23J2215	G05B17, G05B13, H04Q9, G01L3, G01M99
B60K8	812.2705	B62D35	G06F12, G11C13, G11C16, H04R1
C08J2205	790.1235	C08J9	G01N13, G01N15, G01N5, G06F12
C10B31	787.8371	F23G2203	G01L2019, G01V1, G06F9, G11C11, H03K19
B29K2077	766.893	B29L2009	G01N2030, G01N25, G02F1, G01N11, G01N30
B01D9	739.0385	C22B26	G02F1, G01N2030, G01N30, G06Q10, G06Q30
D04H1	729.7817	C08J9	G01N13, G01N15, G01N5, G06F12
C03B19	725.6061	C03B17	G01N27, G01N2021, G01N21, G11B5
C08J2427	697.3836	C08L27	G02F1, G01N27, G01N25, G01N33, G01R31
C22C2026	676.3674	B22F3	G01N27, G01S3, B41J2, G02B6, G02F1
F02G2254	672.6506	F02G1	G01F9, G05B15, G05F1, G06F1, H01S3
C08B16	662.9185	B29B2017	B07C5
C03B2201	662.3126	C03B17	G01N27, G01N2021, G01N21, G11B5
F02P13	633.5146	C10B51	G01V1, G11C11, H03K19, G01L2019
C01B2210	628.6975	C01B21	G05B19, G11B5
F23D2203	626.8618	F23D14	G01F23, G02F1, G06Q30, G06Q50, G01N11
A62C99	623.6007	A62C3	G11B33, G06V20, G01R31, H04N7, G01M99
D01F2	619.5953	B29B2017	B07C5
C08B17	619.1618	B29B2017	B07C5
D01F13	619.1618	B29B2017	B07C5
B02C17	614.6137	B02C21	B07C5, G01F23, G01N35, G06Q30, G01K13
C07F3	611.118	C07F5	G01N33, G01N27, G01N21, G01N2021, G02F1
F01P2060	610.7944	F01P7	H01S3, G01K1, G01N11, G01S19, G01W1
C08J2451	609.5084	C08J2423	G02F1, G02B6, G06F12
C08L51	607.5727	C08L53	G03G15, G02F1, G03G13, G03G9, G06T19
B29K2081	602.6351	B29L2009	G01N2030, G01N25, G02F1, G01N11, G01N30
D10B2201	574.9507	B29B2017	B07C5
B82Y35	572.6666	C01B35	G01N27, G02F1, H01S5
C10G21	569.6333	B01D17	G01N15, G01M1, G01M13, G01N1, G01N2001
F23D17	567.7482	F23K5	H04N11, G06Q30, G06Q50, G09B23, G01F1

## Discussion

Despite the salient trend of energy system digitalization, it is still difficult to identify the R&D direction regarding the convergence between the two complex technology sectors, i.e., the energy sector and the ICT sector. Particularly, in pursuit of sustainability and green growth, LCET innovative agents need to follow ICT development and identify opportunities for digitalization development. In response, our methodology identifies digitalization technology opportunities customized to the current LCET field technology portfolio, so that the LCET innovative agents could potentially increase the possibility of success in digital R&D. In this study, a set of highly recommended LCET-related CPC codes were identified using a collaborative filtering technique. In addition, we assigned the identified CPC codes to different LCET fields based on the current LCET technological portfolio.

Over half of the identified CPC codes belong to the Energy storage field. This result makes sense because, first, the share of Energy storage patents is the largest in LCET. Along with the rapid digitalization trend, more inventions in basic technology might imply more ICT convergence opportunities. Second, our finding is in line with some previous studies concerning the digitalization trend of energy-storage systems. For example, Zhang et al. [3] found that the digitalization of energy storage system had accelerated significantly since 2018; Mejia et al. [44] found that industry research in the energy storage field had been directed toward electric digital data processing for multi-power systems. Moreover, the significantly larger volumes of energy storage patents and digitalization technology opportunities also correspond to previous studies that presented the importance of energy storage digitalization in enhancing system operation and maintenance [17].

Although there were also considerable Solar PV patents applied in the period 2016–2020, none of the identified CPC codes are in that field. This result is consistent with previous studies concerning the nature and digitalization of Solar PV technology. Solar PV, which has a high scale of production, follows the life-cycle pattern of mass-produced goods: early product innovations were followed by a surge of process innovations in solar cell production [45]. The improvement of the energy conversion efficiency and the decrease of solar cell production cost both rely on the advance of basic material technologies. However, the basic technologies may have little interaction with the digital solutions. In this way, given the rapid growth of investments and inventions in solar PV [39], ICT convergence opportunities are still scarce [5].

Note that although few digitalization opportunities in fields such as Solar PV and Wind are identified in this analysis, it does not mean that their digitalization tends to be stagnant. The digitalization of Energy storage is one key implement to support the development of renewable energy technologies. Renewables, such as Solar PV and Wind, are inherently intermittent. It is crucial to have enough flexibility in the power system for reliability and effectiveness when maintaining a high renewable market penetration [1]. Digitally enabled demand response and energy storage are expected to facilitate a higher share of solar PV and wind power and reduce CO_2_ emissions [1, 46].

The identified CPC codes in our analysis illustrate practical R&D directions, which facilitate LCET innovative agents to follow the rapid ICT convergence. For clean combustion field, the identified digitalization opportunities are mainly related to engines and burners. Typical ICT technologies associated with measuring, controlling, and material analyzing, e.g., G01F23 (indicating or measuring liquid level or level of fluent solid material), G05B13 (adaptive control systems), and G01N11 (investigating flow properties of materials; analysing materials by determining flow properties) could provide the potential digital solutions for clean combustion technology. For energy storage field, the identified digital technology opportunities are mainly related to electrode and electrolyte materials and energy storage devices. In addition to applying measuring, controlling, and material analyzing technologies to material preparation, sorting technology may also play a role in improving the overall performance of energy storage material processing, e.g., B07C5 (sorting according to a characteristic or feature of the articles or material being sorted) could be combined with D01F2 (monocomponent artificial filaments or the like of cellulose or cellulose derivatives) for battery separator. Similarly, measuring, controlling, and material analyzing technologies could also work as the digital solutions for material related digitalization opportunities in Fuel cells, Hydrogen, Non-fossil fuel, and Solar PV fields, while computing related technologies, e.g., G06F9 for arrangements for program control, could be involved to improve the overall performance of LCET system.

Besides, policy implications could be derived from the analysis. Given the importance of digitally enabled energy storage, as well as the salient digitalization technology opportunities in the energy storage field, it is necessary to encourage inter-sector R&D activities to foster interdisciplinary inventions. For example, policies or demonstration projects that facilitate the collaboration between energy storage firms and renewable energy firms, such as solar PV and wind power firms, are expected to accelerate LCET digitalization and energy system decarbonization. Moreover, along with the rapid development of emerging digital technologies, such as blockchain, big data, and cloud computing, it is also important for both innovative agents and policy-makers to strengthen the practical applications of digital solutions during product and process innovation, as well as throughout the entire chain of LCET.

## Conclusions

Technology convergence has become increasingly relevant to technology changes, which provides the opportunity window for latecomers’ catch-up and can reshape the competitive landscape, especially with the trend of digitalization. The diffusion of ICT has profoundly impacted social life. In the energy sector, rapid digitalization, especially in the LCET, provides a reliable path for transition to a greener energy system. Given that the trend of digitalization of LCET has been empirically analyzed based on patent data, there are still challenges in identifying the technology opportunities of LCET digitalization, which is of strategic importance for both innovative agents and policy-makers in capturing the forthcoming changes.

To address the challenges, this paper proposes an adapted collaborative filtering using patent data, from the perspective of technology convergence. In this paper, the proposed collaborative filtering is applied to a large LCET patent dataset derived from the United States Patent and Trademark Office (USPTO). Specifically, we first empirically justify the effectiveness of the proposed collaborative filtering in the historical digitalization technology opportunity identification based on LCET patents applied in the period 2011–2015. Then, based on the dataset of 2016–2020, we identify the current digitalization technology opportunities further in the LCET domains. The results show that the LCET digitalization technology opportunities identified through the proposed method are primarily concentrated in the field of Energy storage, which accounts for 16 of the 30 identified CPC main groups. Besides, the identified digitalization technology opportunities are mainly found in the CPC "B. Performing operations; transporting", "C. Chemistry; metallurgy" and "F. Mechanical engineering; lighting; heating; weapons; blasting" Sections.

The proposed method is of high data availability and replicability. Researchers can further apply this method to other technologies to identify technology convergence opportunities. However, there are still some limitations in this paper. For example, the proposed methodology only considers the technical factor that drives the technology convergence, while ignoring potential market factors. Thus, future studies can incorporate dimensions such as market demand to pursue a more comprehensive method.

## Supporting information

S1 Data (ZIP)
